# Por que não Pensar na Estimulação Cerebral Não-Invasiva para Controlar a Pressão Arterial?

**DOI:** 10.36660/abc.20200639

**Published:** 2021-02-19

**Authors:** Fernando Zanela da Silva Arêas, Guilherme Peixoto Tinoco Arêas

**Affiliations:** 1 Universidade Federal do Espírito Santo Depto Fisioterapia Programa de pós graduação em Ciências Fisiológicas VitóriaES Brasil Universidade Federal do Espírito Santo - Depto Fisioterapia, Programa de pós graduação em Ciências Fisiológicas, Vitória, ES - Brasil; 2 Universidade Federal do Amazonas Departamento de Fisiologia ManausAM Brasil Universidade Federal do Amazonas - Departamento de Fisiologia, Manaus, AM - Brasil

**Keywords:** Pressão Arterial, Estimulação Magnetica Transcraniana Corrente Contínua, Estimulação Magnética Não Invasiva/métodos

Em 2007, Ridding e Rothwell^1^ perguntaram seu editorial: “Há um futuro para o uso terapêutico da estimulação magnética transcraniana?”, chamando a atenção para a quantidade de estudos e hipóteses sendo construídas em torno da estimulação cerebral não invasiva (ECNI). De fato, na época, o foco do uso da ECNI era nas doenças neurológicas e psiquiátricas. À medida que o entendimento da fisiologia do sistema nervoso sobre o sistema cardiovascular foi ampliado, outras ideias surgiram. Em sua hipótese, Cogiamanian et al.,^2^ chamaram a atenção para a possibilidade de tratamento da hipertensão arterial utilizando ECNI - estimulação magnética transcraniana repetitiva (EMTr) e estimulação transcraniana por corrente contínua (ETCC). De 2010 até o presente, muitas perguntas foram respondidas sobre os reais efeitos da ECNI, através de pesquisas experimentais e clínicas.^3 , 4^ O entendimento que temos hoje dos efeitos fisiológicos que as técnicas de estimulação cerebral têm nas células neuronais e associando isso ao complexo controle neural do sistema cardiovascular, nos perguntamos: existe a possibilidade dessa abordagem eficaz de tratamento da hipertensão arterial sistêmica? Se pensarmos no baixo custo, fácil adesão do paciente, poucos efeitos colaterais, parece-nos razoável a necessidade de estudos que investiguem essa possível abordagem. Há alguns anos, a relação entre os mecanismos fisiopatológicos da hipertensão arterial e o sistema nervoso central e periférico tem sido mais estudada e pensando nos mecanismos de controle da diminuição e regulação da pressão arterial (PA), várias áreas do cérebro (córtex sensório-motor, pré-córtex-córtex frontal medial e córtex insular) controlam diversas funções, tais como: modulação da resposta autonômica, mecanismos vasomotores somáticos, variações da PA, entre outras.

Estudos têm demonstrado que a ECNI pode influenciar o comportamento autonômico cardíaco, por meio da variabilidade dos intervalos R do eletrocardiograma, favorecendo o aumento da atividade simpática ou parassimpática cardíaca dependendo diretamente do estímulo aplicado.^5^ Aparentemente, a estimulação anódica aplicada à área de controle motor aumenta o tônus simpático, enquanto a estimulação anódica no lobo temporal aumenta o tônus parassimpático (córtex insular).^6 , 7^ Entretanto, pouco foi explorado em resposta à ECNI na atividade simpática arterial. Sabe-se que a PA é controlada pelo débito cardíaco (frequência cardíaca e volume sistólico) e pelo sistema de resistência arterial. A ação central da modulação cardíaca e periférica pelo sistema autônomo através da ECNI pode ser uma viabilidade não farmacológica no controle da PA, com grande plausibilidade. A ação direta na redução da pressão só foi verificada quando a estimulação cerebral profunda foi realizada na região da substância cinzenta periventricular/periaquedutal em humanos.^8^ Porém, em estudos com ETCC realizados em indivíduos normotensos, ela não demonstrou nenhum efeito hipotensor.^9^ Uma luz no fim do túnel para o efeito da ECNI na PA foi verificada com um estudo utilizando ETCC em atletas. Maior hipotensão pós-exercício aeróbio foi encontrada quando a ETCC foi aplicada antes do exercício em atletas, não tendo efeito em indivíduos sedentários.^10^ Porém, o efeito de curto, médio e longo prazo da ECNI em indivíduos hipertensos, principalmente por causas idiopáticas, precisa ser melhor verificado.

Sabe-se que pacientes hipertensos apresentam maior tônus simpático cardíaco e vascular.^2^

Possivelmente, os efeitos cardíacos são mais esperados com a aplicação da ECNI do que os periféricos; portanto, podemos esperar a probabilidade de que a diminuição da resposta simpática em pacientes hipertensos facilite a hipotensão ou melhore o efeito farmacológico e não farmacológico no tratamento clínico.

Diante dessa relação, entre a PA e a atividade cortical, a ECNI parece ser uma ferramenta com bom potencial a ser explorado, pois os efeitos da estimulação cerebral invasiva (estimulação cerebral profunda e estimulação medular) já foram demonstrados com bons resultados no controle da PA.^8^ A redução da PA é vista como uma reação após a estimulação magnética não invasiva. Esta carta chama a atenção para esta hipótese devido aos poucos estudos experimentais e clínicos que testaram esta possibilidade e os estudos clínicos que temos não abordaram de maneira mais incisiva a hipertensão arterial sistêmica.
