# Supplementary material for: Por que não Pensar na Estimulação Cerebral Não-Invasiva para Controlar a Pressão Arterial?
Source: Arq Bras Cardiol. 2021 Feb 19;116(2):349–50. [Article in Portuguese] doi: 10.36660/abc.20200639 (PMC7909986; doi:10.36660/abc.20200639)
Supplement: Translation

# Translation: Why not Think about Non-Invasive Brain Stimulation to Control Blood Pressure?

**Authors:** Fernando Zanela da Silva Arêas, Guilherme Peixoto Tinoco Arêas

**Affiliations:** 1 Brazil Universidade Federal do Espírito Santo - Depto Fisioterapia, Programa de pós graduação em Ciências Fisiológicas, Vitória, ES - Brazil; 2 Brazil Universidade Federal do Amazonas - Departamento de Fisiologia, Manaus, AM - Brazil

**Keywords:** Blood Pressure, Transcranial Magnetic Stimulation, Transcranial Direct Current Stimulation, Transcranial Magnetic Non-Invasive/methods

In 2007, Ridding and Rothwell^1^asked in their editorial: “Is there a future for the therapeutic use of transcranial magnetic stimulation?”, drawing attention to the number of studies and hypotheses being built around non-invasive brain stimulation (NIBS). In fact, at the time, the focus of the use of NIBS was on neurological and psychiatric diseases. As the understanding of the physiology of the nervous system on the cardiovascular system was broadened, other ideas emerged. In their hypothesis, Cogiamanian et al. (2010)^2^drew attention to the possibility of treating arterial hypertension using NIBS - repetitive transcranial magnetic stimulation (rTMS) and transcranial direct current stimulation (tDCS). From 2010 to the present, many questions have been answered about the real effects of NIBS, through experimental and clinical research.^3 , 4^The understanding we have today of the physiological effects that brain stimulation techniques have on neuronal cells and associating them with the complex neural control of the cardiovascular system, we ask ourselves: is there a possibility of this effective treatment approach for systemic arterial hypertension? If we think about the low cost, easy patient adherence, few side effects, it seems reasonable for us the need for studies that investigate this possible approach. For some years now, the relationship between the pathophysiological mechanisms of arterial hypertension and the central and peripheral nervous system has been further studied and thinking of the descending blood pressure (BP) control and regulation mechanisms, various areas of the brain (sensory motor cortex, pre-cortex - frontal medial and the insular cortex) control several functions such as: modulation of the autonomic response, somatic vasomotor mechanisms, variations in BP, among others.

Studies have shown that NBIS can influence cardiac autonomic behavior, through the variability of the R intervals of the electrocardiogram, favoring the increase in cardiac sympathetic or parasympathetic activity, depending directly on the applied stimulus.^5^Apparently, anodic stimulation applied to the motor control area increases the sympathetic tone, while anodic stimulation in the temporal lobe increases the parasympathetic tone (Insular cortex).^6 , 7^However, little has been explored in response to NIBS on arterial sympathetic activity. It is well known that BP is controlled by cardiac output (heart rate and stroke volume) and by the arterial resistance system. The central action of cardiac and peripheral modulation by the autonomic system through NIBS can be a non-pharmacological feasibility in BP control, with great plausibility. The direct action on pressure reduction was only verified when deep brain stimulation was performed in the periventricular/periaqueductal gray matter region in humans.^8^However, in studies with tDCS carried out in normotensive individuals, it failed to show any hypotensive effect.^9^A light at the end of the tunnel for the effect of NIBS on BP was verified in a study using tDCS in athletes. Higher post-aerobic exercise hypotension was found when tDCS was applied before exercise in athletes, with no effect on sedentary individuals.^10^However, the short, medium and long-term effect of NIBS in hypertensive individuals, mainly due to idiopathic causes, needs to be better verified.

It is known that hypertensive patients have greater cardiac and vascular sympathetic tone.^2^

It is possible that cardiac effects are more expected with the application of NIBS than peripheral ones, thereby we can expect the likelihood that the decreased sympathetic response in hypertensive patients facilitates hypotension or improve the pharmacological and non-pharmacological effect in the clinical treatment.

In view of this relationship between BP and cortical activity, NIBS seems to be a tool with good potential to be explored, as the effects of invasive brain stimulation (deep brain stimulation and spinal cord stimulation) have already been demonstrated with good results in the control of BP.^8^The reduction in BP is seen as a reaction after non-invasive magnetic stimulation. This letter draws attention to this hypothesis due to the few experimental and clinical studies that have tested this possibility and the clinical studies that we have, did not address systemic arterial hypertension more strongly.
